# Investigating within-host population diversity of *Cryptosporidium parvum* using BlooMine

**DOI:** 10.1186/s12864-025-12206-4

**Published:** 2025-11-20

**Authors:** Arthur V. Morris, Thomas Connor, Justin Pachebat, Martin Swain

**Affiliations:** 1 School of Biological Sciences, Cardiff University, Cardiff, United Kingdom; 2 Institute of Biological, Environmental and Rural Sciences, Aberystwyth University, Aberystwyth, United Kingdom; 3 Department of Life Sciences, Aberystwyth University, Aberystwyth, United Kingdom

**Keywords:** Cryptosporidium, Genomic, Zoonosis, Disease, Bioinformatics

## Abstract

Investigating multiplicity-of-infection (MOI) in pathogen populations is central to understanding within-host evolutionary dynamics. In *Cryptosporidium parvum*, MOI may play a pivotal role in diversification due to the parasite’s sexually recombinogenic life cycle, which occurs entirely within a single host. Subtyping of *C. parvum* typically relies on variation at short tandem repeat (STR) loci - such as that found in the 60kDa surface glycoprotein gene, Gp60 - but accurate profiling of these regions from next-generation sequencing (NGS) data remains technically challenging. Here, we apply BlooMine, an alignment-free, Bloom filter based tool that isolates STR containing reads via a novel pseudo-alignment strategy robust to structural and sequence variation. Using this approach, we analyse the full publicly available *C. parvum* Illumina dataset to quantify polyclonality at the Gp60 locus. After stringent artefact suppression, we detect strong evidence of in-host diversity across multiple continents and hosts. Cattle samples exhibited a 2.3-fold higher odds of harbouring multiple Gp60 subtypes compared to human samples, a difference that remained significant after adjustment for geography, sequencing depth, and subtype family. Allele co-occurrence analysis revealed closely related pairs likely arising from replication slippage, as well as mutually exclusive combinations suggestive of within-host competition or transmission structuring. Several subtypes traditionally considered host-specific were detected in unexpected host contexts, indicating greater host plasticity than previously assumed. Our results support a model in which STR-driven microevolution, recombination, and polyclonal infection jointly shape *C. parvum* population structure. We propose that MOI may act as a genetic crucible, facilitating subtype diversification within individual hosts. This study represents the largest genomic survey of Gp60 polyclonality to date and provides key insights into the evolutionary and epidemiological dynamics of *C. parvum*.

**Supplementary Information:**

The online version contains supplementary material available at 10.1186/s12864-025-12206-4.

## Introduction

*Cryptosporidium* is a single celled eukaryotic parasite which causes substantial mortality and morbidity worldwide, with it being implicated in over 200,000 deaths annually [1]. Extracting sufficient DNA from clinical samples of *Cryptosporidium* for the purpose of whole genome analysis is a challenge due to the often low faecal mass provided in clinical samples, and low oocyst count of these samples [2, 3]. Despite recent advances being made both experimentally and computationally, there is currently no workflow which allows for routine generation and analysis of whole genome data from clinical *Cryptosporidium* samples [4–6].

Multiplicity of infection (MOI) is a well documented observation within the natural world, and has been reported widely in parasitic protozoan infection. In 1999, Lord et al. reported that the majority of adults infected with *Plasmodium falciparum* were host to more than five distinct strains [7]. MOI may arise by two principal processes: infection by multiple strains; and in-host divergence into genetically distinct sub-populations. The presence of multiple clonal populations of a parasite in a single host (referred to as ‘polyclonality’) has a significant impact on their incidence, transmission, and virulence, factors of enormous importance to public health. An incomplete understanding of parasite polyclonality leads to potentially inaccurate assumptions about the epidemiology, clinical presentation and outlook of the infection. The virulence experienced by a host which exhibits polyclonality is a product of the interactions between the different clonal populations, resulting in an overall virulence of anywhere from greater than the most virulent to less than the least virulent, depending on various biological factors [8, 9]. Efforts have been made to identify general patterns when ecological and evolutionary theory are applied to within-host dynamics, leading to the generalisation that ‘basic ecological rules govern the outcome of co-infection across a broad spectrum of parasite taxa’ [10]. The investigation into the impact of MOI on a host relies on the accurate and reliable detection and discrimination of the clonal component populations of the mixture. With modern methods of DNA isolation and purification, and the fall in price of using high-throughput Next-Generation sequencing (NGS) technologies to perform whole genome sequencing (WGS), investigating MOI by mining WGS reads has become a more feasible approach [2, 3]. Furthermore, it allows for unrestricted investigation of local heterogeneity at any position within the genome, something which is simply not possible to carry out using conventional molecular wet-lab techniques. It is both biologically plausible (due to unrestricted sexual recombination between sub-populations), and there is strong evidence, that mixed-infections can give rise to novel strains of *Cryptosporidium* spp., which may be genotypically and phenotypically distinct from their parents, representing a serious challenge to public health control services which rely on epidemiological surveillance [11–14]. It is therefore essential that polyclonality can be characterised in order to improve epidemiological surveillance, which is essential in outbreak investigation and subsequent interruption of transmission - a critical component in the fight against cryptosporidiosis and other parasitic diseases.

Alignment-free sequence analysis has the capacity to resolve the significant bottleneck between data generation and analysis which has been well documented in ‘omics research, as it may be both faster and more computationally efficient than sequence alignment based approaches [15]. This allows for the development of tools which can mine information from raw sequencing reads, obviating the significantly time-consuming and computationally expensive task of assembly. There are a great many tools which employ alignment-free methods to identify nucleotide and protein sequence similarity, developed to resolve problems in many disciplines within Bioinformatics. However, the majority of the tools developed to resolve these from pathogen clinical data utilise single nucleotide polymorphism (SNP) data, for which variant callers are highly optimised. Variable Number Tandem Repeat (VNTR) loci have been regions subject to heavy interrogation, and have been used to define subtypes and variants in many pathogen species [16]. Despite great advances in the use of NGS data for assembly-free genomic analysis, low concordance of variant calling pipelines has been reported, and such pipelines are often incapable of resolving VNTR repeat subunit copy number even across small VNTRs which can be captured in a single read, which remains a troubling issue [17].

Here we present BlooMine: a novel raw read mining tool developed to facilitate quick and computationally efficient local analysis of sequences captured within raw reads generated by whole or partial genome sequencing projects. It utilises Bloom filters, a highly space efficient probabilistic data structure, and a novel pseudo-alignment algorithm to perform set membership queries and infer sequence homology [18]. We use BlooMine to investigate MOI in clinical samples of *Cryptosporidium parvum*, demonstrating that it can be used to perform both *in silico* subtyping from NGS reads, and local genomic heterogeneity analysis, facilitating both informed epidemiological investigations in public health, and robust hypothesis testing critical to the development of novel approaches of transmission interruption.

## Materials and methods

### Implementation

BlooMine is written using C++ and Python 3. It consists of two modules:

1. BlooMine_screen: Screen a FASTQ dataset for reads which contain a target sequence.
2. BlooMine_demix: Analyse reads for target sequence heterogeneity.

The BlooMine_screen module consists of 3 steps:

- BlooMine_gen: Generate a Bloom filter from the target sequence.
- BlooMine_FPscreen: Carry out a first pass screen of reads using the target Bloom filter.
- BlooMine_SPaln: Carry out a second pass target sequence pseudo-alignment step.

#### BlooMine_gen

A target sequence, *T* is used to generate a *k*mer array, $K_T^k$. This is then passed through hash functions to generate a bit signature for *T*, $B_t$. The number of hash functions can be adjusted to meet user type-I and type-II error rate requirements.

#### BlooMine_FPscreen

Reads within the FASTQ file are screened against the Bloom filter, $B_t$, generated during the BlooMine_gen process to detect the presence of the target sequence. This step constitutes the first pass screening. A *k*mer array is generated from each read within the FASTQ, which is then hashed using the Bloom filter hash functions. The subsequent bit signature generated by this hashing process is screened against $B_t$ to infer set membership. The detection of a hit increments the score by 1. If the overall score for the read exceeds the *k*mer intersection threshold, sequence similarity is inferred and the read returned for further processing. Trivially (without a description of Bloom filter functionality) the set of all reads which exceed the *k*mer intersection threshold is therefore

$$\begin{aligned} R' = \{ r \in R\ \big \vert \ |K_T^k \cap K_r^k| \ge \theta \} \end{aligned}$$

where $K_r^k$ is a *k*mer array of the read sequence, *T* is the target sequence, and $\theta$ is *k*mer intersection threshold.

#### BlooMine_SPaln

BlooMine_SPaln filters reads using a novel soft-alignment algorithm (Algorithm 1) based on positional *k*-mer mapping. For each read in the filtered set $R'$, the positions of all matching *k*mers are identified and used to construct a sparse positional vector aligned to the read.

**Figure:**
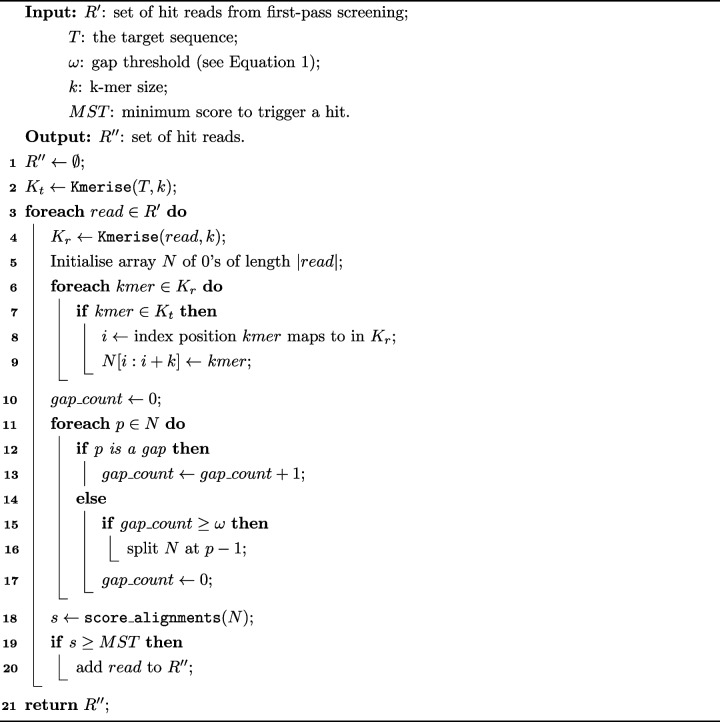
**Algorithm 1** BlooMine SPaln Second-pass screen: Alignment-free alignment inference

From this vector, gapped alignment regions are inferred by grouping together adjacent *k*mer hits that are separated by no more than a threshold number of positions. These are defined as subalignment chunks. Each chunk is scored using a gap-sensitive function to determine the optimal alignment region.

The gap threshold $\omega$ is defined as:

1 $$\begin{aligned} \omega = \left\lceil \frac{hk - g}{n} \right\rceil \end{aligned}$$

This value is chosen such that a single matching *k*mer followed by a gap of size $\omega$ results in a total alignment score near zero, thereby discouraging long, sparse alignments.

Each chunk *c* is then scored using the following scoring algorithm. Let chunk $c=\{c_0,c_1,...,c_{|c|-1}\}$ be a sequence of aligned positions (indexed by position in the read), we define the score of the chunk $s_c$ as:

2 $$\begin{aligned} s_c = \sum \limits _{i=0}^{|c|-1}\delta (c_i,c_{i-1}) \end{aligned}$$

Where $\delta (c_i,c_{i-1})$ is defined as:

3 $$\begin{aligned} \delta (c_i,c_{i-1}) = \left\{ \begin{array}{ll} +h & \text {if } c_i \in K_T \\ -g & \text {if } c_i \notin K_T \wedge c_{i-1} \in K_T \\ -n & \text {if } c_i \notin K_T \wedge c_{i-1} \notin K_T \end{array}\right. \end{aligned}$$

Where:

*k* = the size of each *k*mer (default=7).

*h* = the value the score is incremented by when a hit is triggered (10.0).

*g* = the value the score is penalised by when a gap is opened (15).

*n* = the value the score is penalised by when a gap is extended (7.0).

$c_i$ = position *i* within the aligned chunk *c*.

$K_T$ = the *k*mer array of target sequence *T*.

**Fig. 1:**
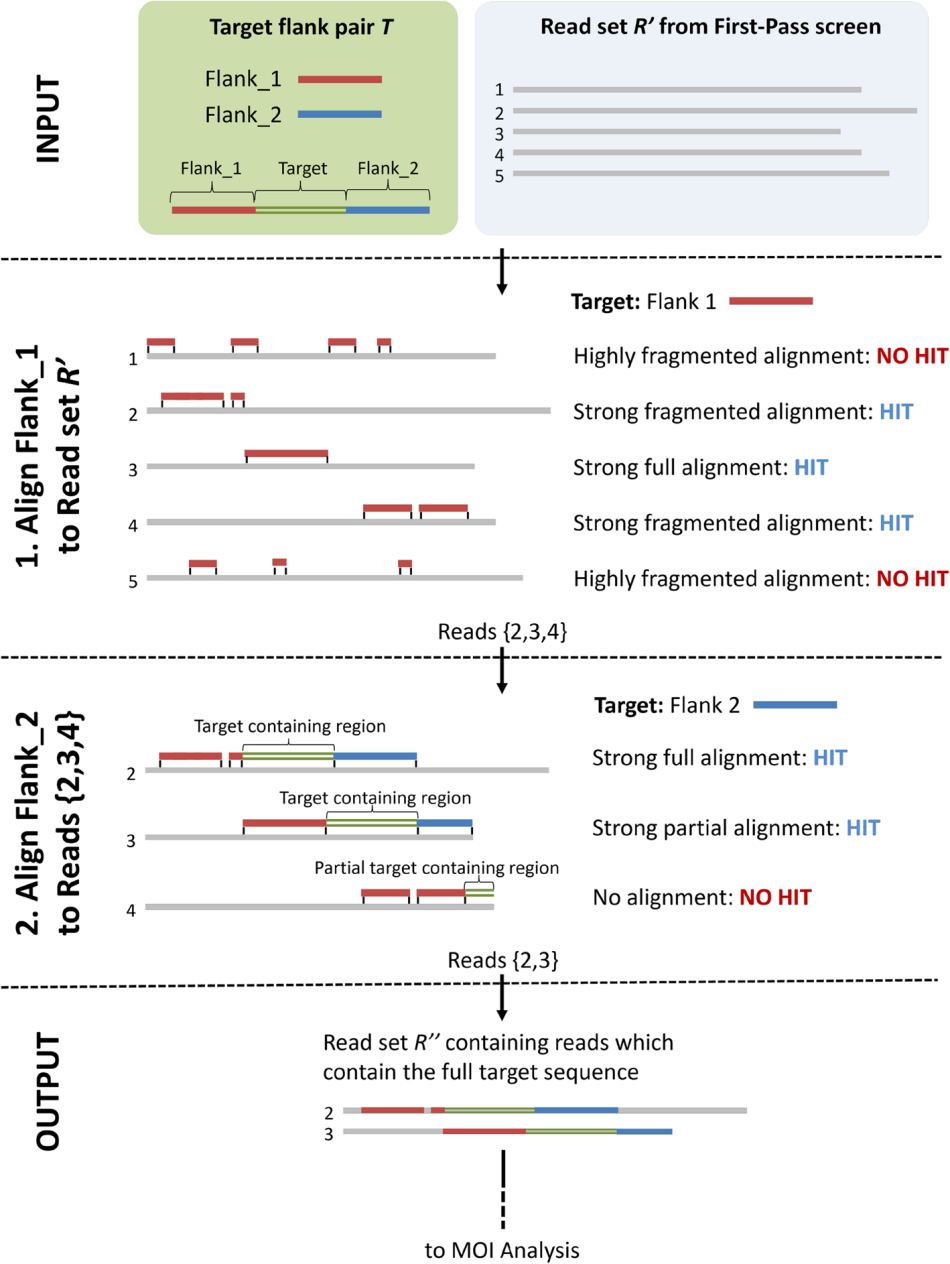
Workflow for identifying target regions in short reads

**Fig. 2:**
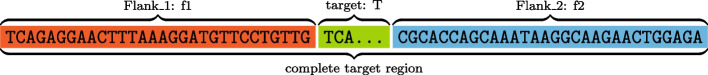
A model of a target sequence, showing the target region consisting of a TCA repeat region, and flanking regions. Where... refers to an arbitrary number of the preceding repeat motif

Alignment regions are split at any gap of size $\ge \omega$. All contiguous sub-sequences of alignment chunks (i.e. $\mathcal {O}(n(n+1)/2)$ combinations for *n* chunks) are evaluated using the scoring function. The highest-scoring alignment is selected. If this score exceeds the minimum score threshold *MST* (see Minimum score threshold section), the read is deemed to contain the target sequence. Figure 1 presents a graphical representation of this process using a full target region consisting of two flanking regions and a polymorphic STR locus (see Fig. 2 for an example model of an STR target region).

#### Minimum score threshold

Due to the polymorphic nature of certain genomic regions (e.g. VNTRs), traditional alignment algorithms such as Smith-Waterman and Needleman–Wunsch often fail to produce reliable results due to their assumption of globally consistent match/mismatch patterns [19, 20]. These methods are not robust to high densities of SNPs, insertion/deletion events (INDELs), or rearrangements.

To address this, we propose an approximate alignment scoring scheme based on *k*-mer matches across sliding windows. The key insight is that mismatches often cluster, particularly in low-complexity and repetitive regions, rather than present as uniformly distributed across a sequence.

Let *s* be a quest sequence of length |*s*|, and let $\kappa$ be the expected mismatch spacing (i.e. the average number of bases between mismatches). The mismatch rate is then defined as $\epsilon =\frac{1}{\kappa }$, and the expected number of mismatches in *s* is $|s| \cdot \epsilon$.

We define a sliding window of size $k+\kappa -1$. The expected number of mismatches per window, *e* is therefore

4 $$\begin{aligned} e=\frac{k+\kappa -1}{\kappa } \end{aligned}$$

For example, with $k=5$ and $\kappa =10$, a window spans 14 bases and is expected to contain 1.4 mismatches.

Let $w=\frac{|K_t|}{k+\kappa -1}$ be the number of such windows over the target sequence. The total expected mismatch count, *M*, is

5 $$\begin{aligned} M=e(w-1) \end{aligned}$$

The minimum alignment score is then calculated as

6 $$\begin{aligned} MST=h\cdot |K_t|-wg+nM \end{aligned}$$

This novel alignment algorithm uses concepts from both alignment and alignment-free sequence analysis approaches to resolve alignments from sequences which have undergone rearrangement or INDEL events, since regions flanking highly polymorphic VNTR regions may be poorly conserved. Furthermore, alignments are carried out on small sequences (single reads) potentially many thousands of times, which may render conventional hard-alignment approaches less efficient.

#### BlooMine_MOI

BlooMine functionality can be leveraged to investigate the presence of multiple alleles within a single FASTQ file, which may indicate MOI in clinical pathogen samples. Reads are filtered in two steps using probe sequences flanking the target region as proxy-targets. First, reads containing flank 1 are identified within the full dataset. Second, the resulting subset is further filtered for reads containing flank 2. The output is the set of reads which contain both flanks, and therefore the target region. These reads are then used to calculate the level of heterogeneity in the target region.

Probe sets for three major *C. parvum* Gp60 families (IIa, IId, IIc) were developed using sequences flanking the STR region. These probes were determined to cover a large portion of the *C. parvum* subtype space. To account for probe cross-matching between Gp60 families, the major Gp60 family was heuristically determined using probe set which yielded the largest number of read hits. Instances of multiple Gp60 family hits were handled using bespoke scripts which determined the best matching probe pair for each read.

### MOI investigation of *C. parvum*

A dataset of all publicly available *Cryptosporidium parvum* (n=667) NGS samples was utilised in this study. Samples which were a product of artificial infection, or lab strains, were filtered out (n=38). The remaining samples (n=629) were screened using the highly polymorphic Gp60 microsatellite locus as a target. These samples were taken from Europe, North America, Africa, and Asia, and from 7 hosts: Cattle (*Bos taurus*), Human (*Homo sapiens*), Sheep (*Ovis aries*), Goat (*Capra hircus*), Water Buffalo (*Bubalus bubalis*), Bactrian camel (*Camelus bactrianus*) and Mouse (*Mus musculus*). The BlooMine MOI pipeline was run on each of these samples using sequences taken from conserved regions flanking the Gp60 locus.

BlooMine was executed using a false-positive rate of 0.0001, a FP-screen threshold of 35.0 and a SP-screen threshold ($\kappa$) of 4.0. All data were analysed on a laptop running Ubuntu v22.04.5, with 64Gb memory and using 12 threads.

#### Gp60 subtyping of *Cryptosporidium*

Subtyping of *Cryptosporidium* involves interrogation of a highly polymorphic VNTR (serine repeat) region within the Gp60 surface glycoprotein gene on chromosome 6 [21]. Due to its highly polymorphic nature in *C. parvum*, it has been used to perform epidemiological investigations in this species for many decades. Subtypes are elucidated by determining the fragment length and sequence content of the Gp60 allele (a variant of a target locus).

Here, allele naming follows a convention analogous to that used when reporting the Gp60 subtype of *Cryptosporidium*, which was thoroughly described by Robinson * et al* [22, 23]. A, G, and R are followed by integers representing the numbers of TCA, TCG and terminal ACATCA counts respectively. This yields an allele code like ’A15G2R1’. Gp60 family is determined by the sequence content of the Gp60 gene, and takes the form of a latinised alphanumeric code (e.g. IIa) which indicates both the species and Gp60 family of the sample, whereby the two major species, *C. hominis* and *C. parvum* are designated I and II respectively. This yields a full Gp60 subtype code which indicates the family and the allele present in any given sample (e.g. IIaA15G2R1). However, since the full Gp60 region was not utilised in determining the Gp60 family in this study (due to limitations of read length), we present the probe which exhibited the highest fidelity with each allele after the allele name. For example A15G2R1-IIa would refer to the A15G2R1 allele extracted using probes designed against sequences from Gp60 family IIa. This is done to distinguish it from the conventional nomenclature and ensure that the Gp60 family designations are not taken as definitive.

#### Allele filtering and PCR stutter artefact correction

To accurately characterise the allelic composition of STR loci from sequencing data, particularly the *Gp60* locus, we employ a two-stage filtering process designed to exclude sequencing noise and PCR stutter artefacts.

***Allele Filtering***

If we consider the set of all reads which have been determined to contain a target region by BlooMine as $R''$, then we presume that all variants of the target sequence (’alleles’) are embedded within this read set. An allele count vector is computed for each allele within $R''$ as

7 $$\begin{aligned} \mathcal {C}_{R''} = \left[ c_1, c_2, \dots , c_n \right] \end{aligned}$$

where $c_i$ is the number of reads in $R''$ assigned to allele $a_i$ (the allele count of $a_i$), and *n* is the total number of distinct alleles identified. From this, we define a normalised frequency vector:

8 $$\begin{aligned} \mathcal {F}_{R''} = \left[ f_1, f_2, \dots , f_n \right] = \left[ \frac{c_1}{\sum _{j=1}^n c_j},\; \frac{c_2}{\sum _{j=1}^n c_j},\; \dots ,\; \frac{c_n}{\sum _{j=1}^n c_j} \right] \end{aligned}$$

where $f_i$ is the relative frequency of allele $a_i$ across all reads in $R''$ (the allele frequency of $a_i$). The major allele is defined as the allele with the highest frequency in $\mathcal {F}_{R''}$, and minor alleles are all other alleles.

For example, suppose we have 40 reads which fully cover the *Gp60* region, including its flanking regions. This ensures that the STR locus is not clipped and allows for confident allele calling. The total allele count at this locus is therefore $N = 40$, with observed counts:

$$\begin{aligned} \mathcal {C} = \left[ \begin{array}{l} a_1 : 20 \\ a_2 : 10 \\ a_3 : 6 \\ a_4 : 4 \end{array} \right] \end{aligned}$$

The corresponding frequency vector is:

$$\begin{aligned} \mathcal {F} = \left[ \begin{array}{l} a_1 : 0.5 \\ a_2 : 0.25 \\ a_3 : 0.15 \\ a_4 : 0.10 \end{array} \right] \end{aligned}$$

The major allele is the most frequent, in this case $a_1$, and all others are considered minor alleles. We then apply a naive hard-filter using a minimum count threshold $T_c$ and frequency threshold $T_f$. Alleles that fall below either threshold are removed. If we assign the naive hard-filtering parameters in this examples as $T_c=3$ and $T_f=0.15, a_4$ is filtered out due to its low frequency even though its count exceeds the minimum. This dual-threshold approach is functions to remove noise in both low and high depth scenarios: in high-depth datasets (e.g., $N = 1000$), noise may exhibit high counts but low frequency; in low-depth datasets, even a single erroneous read can represent a substantial proportion.

***PCR Stutter Filtering***

To further reduce artefacts arising from PCR amplification, we apply an additional stutter filter based on known dynamics of replication slippage in STR regions [24]. The most common stutter artefact is a loss of a single repeat unit (back-stutter; *L*1), followed less frequently by a loss of two repeat units (*L*2). Forward stutter and larger contractions are rare [25]. These stutter products are not sequencing errors, but true sequences generated from mis-amplified templates. We account for this by comparing the frequencies of minor alleles to that of the presumed template (major allele). For alleles $a_j$ which are one or two repeat units shorter (thus potential *L*1 or *L*2 artefacts) than the major allele, $a_M$, we compute a stutter ratio:

9 $$\begin{aligned} s_j = \frac{f_j}{f_M} \end{aligned}$$

where $f_j$ and $f_M$ are the frequency of $a_j$ and $a_M$ respectively. We then assign a stutter ratio threshold for *L*1 artefacts ($R_1$), and an *L*2 stutter ratio threshold of $R_2=R_1/2$. *L*1 and *L*2 candidates are considered artefacts if $s_j<R_1$ or $s_j<R_2$ respectively, and thus filtered out [26, 27]. For all other alleles (not plausible stutter products), only the naive filtering thresholds apply.

Continuing the example, suppose the filtered allele set is:

$$\begin{aligned} \left[ \begin{array}{l} a_1 : 20 \\ a_2 : 10 \\ a_3 : 6 \end{array}\right] \end{aligned}$$

with frequencies:

$$\begin{aligned} \left[ \begin{array}{l} a_1 : 0.5 \\ a_2 : 0.25 \\ a_3 : 0.15 \end{array}\right] \end{aligned}$$

Assume the following STR sequences:

- $$a_1$$: TCATCATCATCATCGTCG
- $a_2$: TCATCATCATCA (2 repeat unit loss)
- $a_3$: TCATCATCATCATCG (1 repeat unit loss)

Allele $a_2$ is a candidate *L*2 stutter, $a_3$ is a candidate *L*1 stutter. Normalising the frequencies using Eq. 9 gives us:

$$\begin{aligned} s_{a_2} = \frac{0.25}{0.5} = 0.5, \quad s_{a_3} = \frac{0.15}{0.5} = 0.3 \end{aligned}$$

If we assign aggressive stutter threshold parameters of $R_1 = 0.34$ (thus $R_2 = 0.16$), $a_3$ is filtered ($0.3 < 0.34$), but $a_2$ is retained ($0.5> 0.16$), yielding the final filtered set:

$$\begin{aligned} \mathcal {C'} = \left[ \begin{array}{l} a_1 : 20 \\ a_2 : 10 \end{array}\right] , \quad \mathcal {F'} = \left[ \begin{array}{l} a_1 : 0.667 \\ a_2 : 0.333 \end{array}\right] \end{aligned}$$

#### Polyclonality rate in cattle and humans

Allele calls from the Gp60 locus were filtered to suppress PCR-stutter and low-support artefacts using a pre-specified parameter set: $L1 = 0.15, L2 = 0.075$ (i.e., $L2 = L1/2$), minimum allele count $= 3$, and minimum allele frequency $= 0.05$. Samples from hosts other than cattle (*Bos taurus*) or human (*Homo sapiens*) were excluded. A sample was classified polyclonal if $\ge 2$ alleles passed all filters; otherwise monoclonal.

**Table 1:** The host and continent stratified dataset used to conduct the CMH test. Values are given as: monoclonal count (polyclonal count)

	Human	Cattle	Continent total
Europe	62 (3)	77 (11)	139 (14)
North America	163 (13)	44 (8)	207 (21)
Total	225 (16)	121 (19)	346 (35)

To control for geographic confounding and mitigate Simpson’s paradox, analysis was stratified by continent. Continents with fewer than two samples in either host were excluded *a priori*. The final analysis set comprised $n=381$ cattle and human samples from Europe ($n=153$) and North America ($n=228$), of which 346 were monoclonal and 35 were polyclonal (see Table 1).

Within each retained continent, a $2\times 2$ table (host $\times$ poly/monoclonal sample count) was constructed and per-continent odds ratios (ORs) for polyclonality (cattle vs. human) computed. This stratification controls for uneven geographic distribution, and mitigates Simpson’s paradox. Mantel-Haenszel pooled OR ($OR_{MH}$) with $95\%$ confidence intervals were obtained across continents, and the null hypothesis $H_0:OR_{MH}=1$ tested using the Cochran-Mantel-Haenszel (CMH) test (two-sided, $\alpha =0.05$).

Consistency of stratum specific *OR*s across continents was evaluated with the Breslow-Day (BD) test for homogeneity of stratum-specific ORs, and results interpreted under the criterion that BD $p<0.10$ indicates heterogeneity. Pooled results were interpreted with caution when heterogeneity was detected.

To assess the robustness of hypothesis testing under different filtering parameters, an exploratory threshold sweep over minimum allele count $= 1-30$ and $L1 = 0.01-0.30$ (with *L*2 fixed at $\frac{L1}{2}$, and minimum allele frequency fixed at 0.05) was carried out. For each grid point, the continent-stratified CMH analysis was repeated and summarised by constructing a heatmap from $OR_{MH}$ with CMH *p*-values overlaid. This sweep is descriptive, with all inferences relying on pre-specified thresholds of $L1=0.15$ and minimum allele count = 3, and as such no multiplicity adjustments were carried out.

To probe additional predictors, a multivariable logistic regression was fit with polyclonality as the outcome and covariates: host, continent, sequencing depth (summed Gp60 allele counts across each sample), major-allele length, and Gp60 family (IIa vs IId, IIc excluded due to zero polyclonal samples).

#### Allele co-occurrence

To investigate allele co-occurrence and mutual exclusivity within polyclonal datasets, samples were stratified by continent of origin, and allele pairs with geographical contiguity—defined as co-presence within at least one continent—were tested using the Cochran–Mantel–Haenszel (CMH) procedure. This approach controls for confounding due to uneven geographic distribution, yielding pooled odds ratios and p-values across strata, with inclusion restricted to allele pairs exhibiting geographic contiguity (i.e. allele pairs occurring within at least one shared continent) and meeting a minimum per-continent occurrence threshold of three samples per allele. The Breslow-Day test was used to detect heterogeneity in odds-ratio across different continents. Fisher’s exact test was not applied due to its sensitivity to highly abundant alleles, which can lead to inflated estimates of both exclusivity and co-occurrence signal strength in globally unstratified datasets.

## Results

### Dataset summary

Of the 629 *C. parvum* genomic NGS datasets used, 592 fully covered the Gp60 locus with at least 3 reads and a minimum allele frequency of 0.05. Under the first pass filter only (without stutter filtering), 398 were monoclonal and 194 were polyclonal, exhibiting at least 2 alleles at the Gp60 locus.

**Fig. 3:**
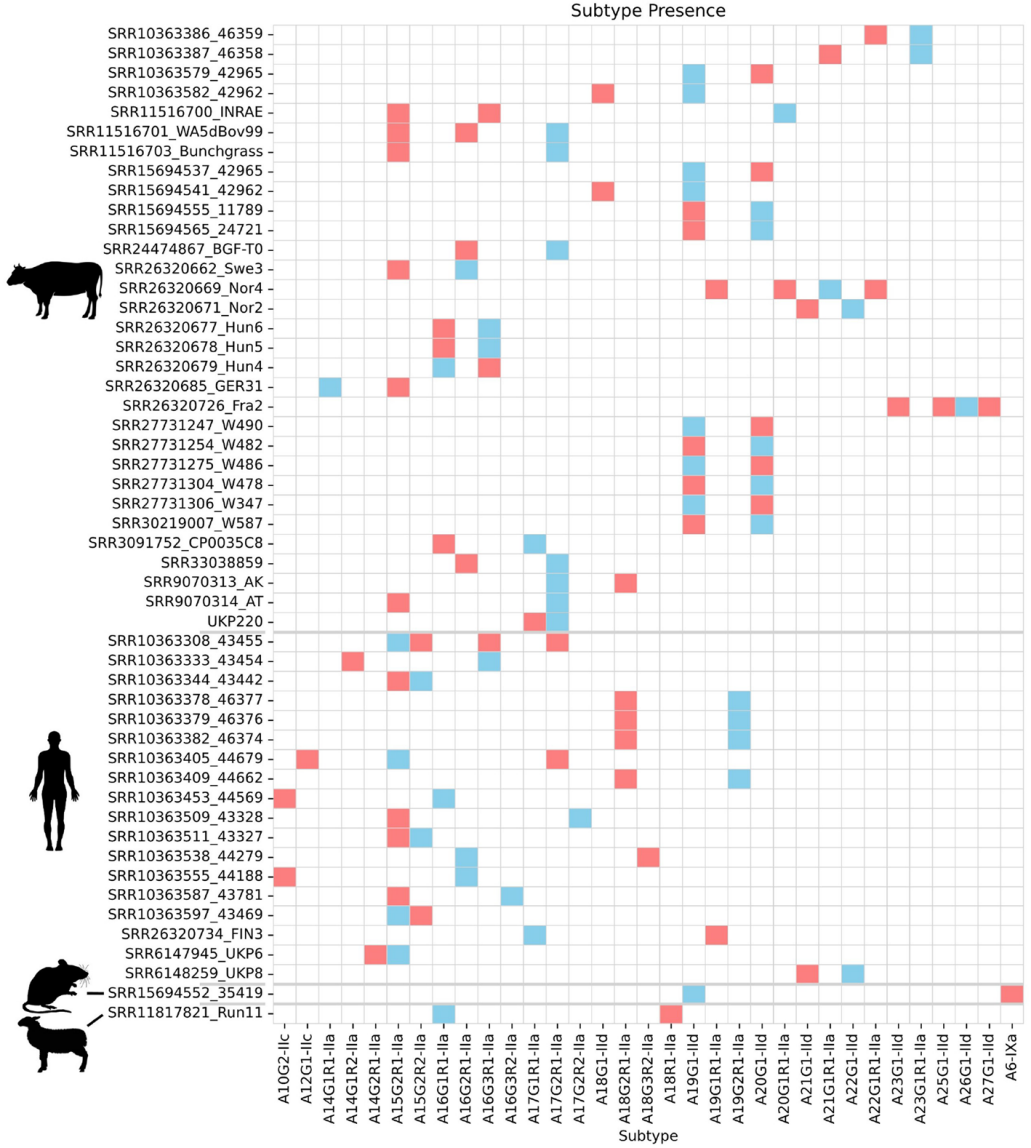
Major/minor allele presence across all 51 samples which were resolved as polyclonal after applying a 0.15 stutter filter. Blue = major allele; Red = minor allele

**Fig. 4:**
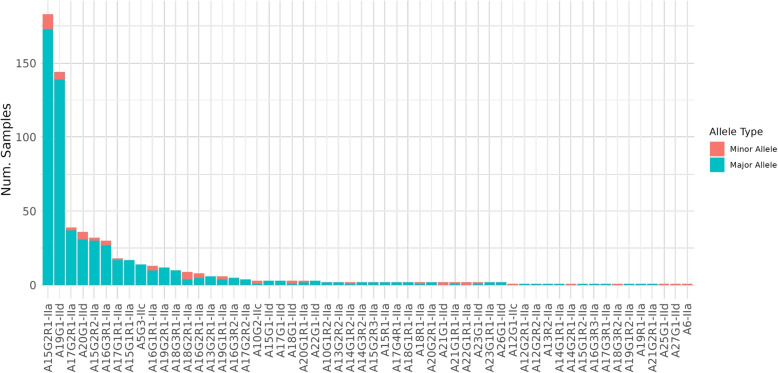
The number of samples which exhibit each allele as major or minor allele

### Polyclonality prevalence

An *L*1 stutter filter of 0.15 was used to investigate allele co-occurrence and host/geographical distributions. Using this stutter filter, 51/592 ($8.6\%$) polyclonal samples were resolved: 18 human, 31 cattle, and one each from sheep and mouse (see Fig. 3). All polyclonal samples were from North America ($n=23$), Europe ($n=15$) and Asia ($n=13$).

In total, 54 unique alleles were detected across clonal and polyclonal samples (Fig. 4); eight occurred only as minor alleles (A14G1R1-IIa, A17G2R1-IIa, A20G1R1-IIa, A22G1-IId, A23G1-IId, A23G1R1-IIa, A25G1-IId, A26G1-IId).

A15G2R1-IIa was the most common allele in the entire dataset ($n=183$) both as a major allele ($n=158$) and minor allele ($n=11$). It was the most frequent major allele in human ($n=109$), sheep ($n=5$) and goat ($n=5$) samples, and the second most common allele in cattle ($n=64$). A15G2R1-IIa comprised $28\%$ of all alleles detected across the dataset and $44\%$ of human samples. The most common major allele in cattle was A19G1-IId ($n=141$) was found in $45\%$ of cattle samples. This allele was also found in $80\%$ ($n=143$) of all Asian samples, which were dominated by Chinese cattle samples, and a single North American sample.

Multiple host-specific alleles were found in humans ($n=18$), cattle ($n=10$), sheep ($n=2$), and mouse ($n=1$). Six of these host-specific alleles were only detected in polyclonal human (A10G2-IIc, A12G1-IIc*, A14G2R1-IIa*, A16G3R2-IIa, A17G2R2-IIa, and A18G3R2-IIa*) and seven in cattle (A14G1R1-IIa, A21G1R1-IIa, A22G1R1-IIa*, A23G1R1-IIa, A23G1-IId*, A25G1-IId*, A27G1-IId*) samples, of which six presented only as minor alleles (denoted by *).

Four samples showed cross-family polyclonality. Three were North American human infections with concurrent IIa and IIc alleles (including one IIa/IIa/IIc triple mixture). The fourth case was a mouse sample in which an atypical allele was recovered using the IId probe set. Subsequent alignment of this cryptic allele against a curated database of Gp60 sequences identified its closest match as *Cryptosporidium tyzzeri*, subtype family IXa.

### Host differences in polyclonality

After filtering, within-continent *OR*s (cattle vs. humans) were 3.23 ($95\%$ CI $0.87-12.02$) in Europe and 1.8 ($0.72-4.49$) in North America, corresponding to polyclonality rates of 0.116 vs. 0.039 and 0.140 vs. 0.083, respectively. The Mantel–Haenszel pooled estimate across continents was $OR_{MH} = 2.26$ ($95\%$ CI $1.08-4.71$; CMH $p = 0.048$), with no evidence of heterogeneity (BD $p = 0.467$). Human IIa/IIc co-infections were observed only in North America and therefore account for the lower continent-specific OR observed there. Cattle had $\sim 2.3$-fold higher odds of harbouring a polyclonal *C. parvum* infection than humans after conditioning on continent.

**Fig. 5:**
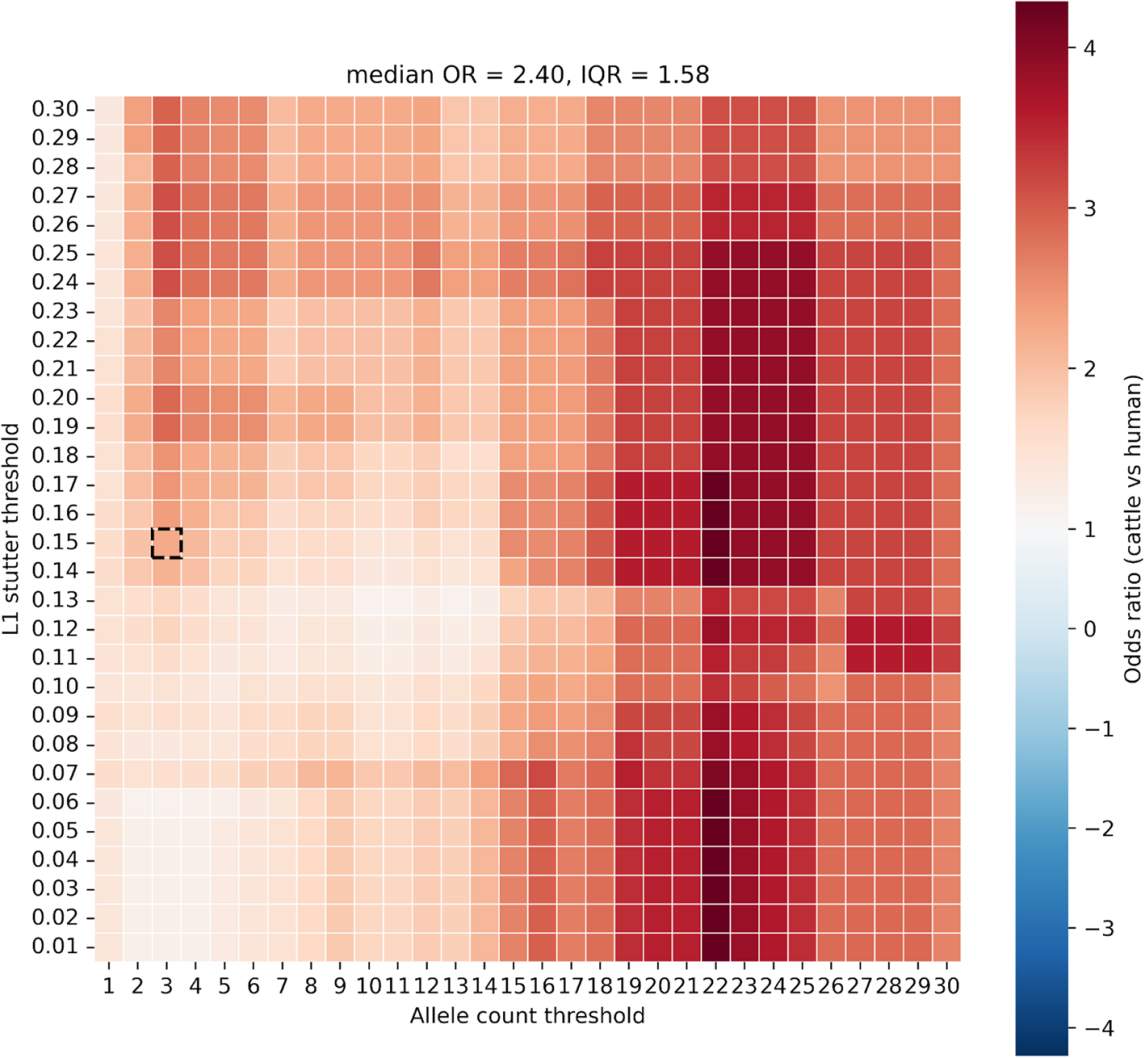
Signed log odds ratios from a continent-stratified Cochran–Mantel–Haenszel test comparing the frequency of polyclonality in cattle versus human samples across a grid of allele count and stutter filter ($L_1$) thresholds. This parameter sweep was conducted to evaluate the robustness of the observed host effect. The black box denotes the pre-specified filtering regime used in all inferential analyses. *OR* = odds ratio; *IQR* = interquartile range

In the multivariable logit model, the host effect remained significant after adjustment for continent, major-allele length, depth, and Gp60 family (adjusted *OR* for cattle vs. human $\approx 2.69$; $95\%$ CI $1.21-5.95$; $p = 0.015$). Sequencing depth ($p = 0.253$), continent (North America vs Europe $p = 0.266$) were not associated with polyclonality in this model. Major-allele length showed a positive association (per-unit $OR = e^{0.1421} \approx 1.15, 95\%$ CI $1.08-1.23$; $p < 0.001$); interpreted over the interquartile range, this corresponds to $OR_{IQR} \approx 2.43$. We consider plausible biological and technical explanations for this signal in the Discussion. Gp60 family ($p=0.729$) was not associated with polyclonality, however, because only IIa and IId were observed across both continents (IIc was only detected in North American samples), the family contrast is sparse and should be interpreted cautiously.

Robustness to filtering choices was evaluated with a parameter sweep over minimum allele count ($1-30$) and *L*1 threshold ($0.01-0.30$; *L*2 fixed at *L*1/2, min $AF = 0.05$). Across a plateau surrounding the pre-specified thresholds (min count $\ge 3, L1 \ge 0.12$), $OR_{MH}$ consistently exceeded 1, with many settings reaching CMH $p < 0.05$ (Fig. 5). As expected, very lenient thresholds (min count $< 3$ and/or $L1 < 0.05$) diluted the signal, consistent with increasing stutter noise rather than a change in the underlying association. Together, these analyses support a continent-adjusted excess of polyclonality in cattle relative to humans, and show that the conclusion is stable to reasonable choices of filtering parameters.

### Allele distribution and co-occurrence

Within-sample allele associations were tested across 140 allele pairs using a continent-stratified CMH model (colour = signed $-log10$ p, bubble size = |*log*2(*OR*)|). Three pairs showed statistically significant positive associations (co-occurrence): A19G2R1-IIa with A18G2R1-IIa ($|log2(OR)|=6.96$), A10G2-IIc with A16G2R1-IIa ($|log2(OR)|=4.80$), and A16G2R1-IIa with A17G2R1-IIa ($|log2(OR)|=2.87$). The latter reflects a moderate effect size. The cross-family association between A10G2-IIc and A16G2R1-IIa is likely a result of the low abundance of A10G2-IIc in combination with its tendency to present as a minor allele within this dataset. In contrast, several pairs exhibited significant negative associations (mutual exclusivity), notably A5G3-IIc with A15G1R1-IIa ($|log2(OR)|=-5.70$), A19G1-IId with A20G1-IId ($|log2(OR)|=-8.13$), and A15G1R1-IIa with A20G1-IId ($|log2(OR)|=-4.39$). The highly prevalent A15G2R1-IIa showed many statistically significant exclusions with other alleles; however, most of these had small effect sizes (small bubbles indicating low |*log*2(*OR*)|).

**Fig. 6:**
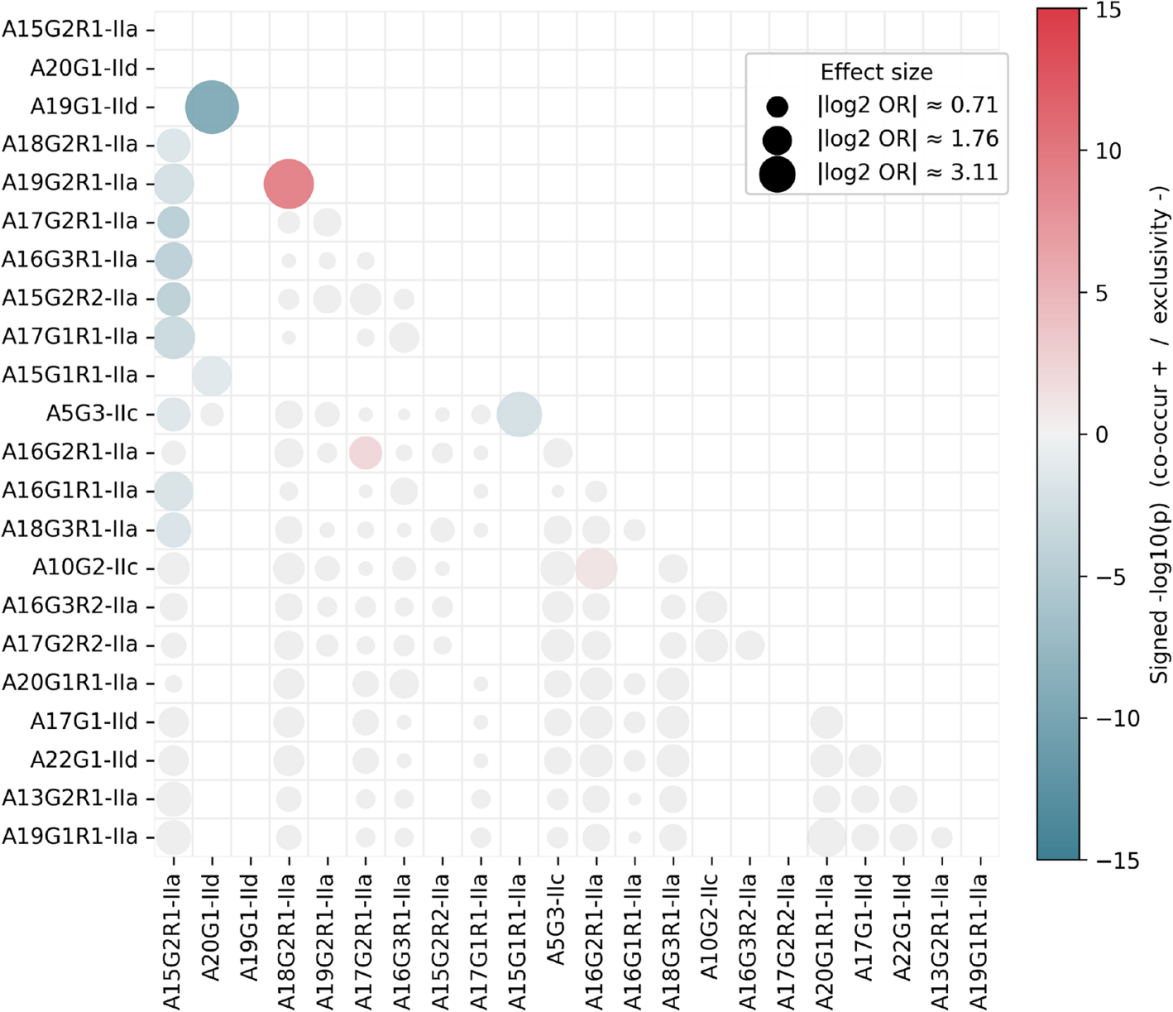
Allele associations conditioned on continent using the Cochran-Mantel–Haenszel (CMH) test. Colour = signed $-log10($one-sided *p*); red indicates positive association (co-occurrence), blue negative (mutual exclusivity). Bubble area is proportional to $\mid log2(OR)\mid$ where *OR* is the CMH pooled odds ratio. Grey bubbles mark non-significant pairs ($p \ge 0.05$)

## Discussion

### Cattle are more likely to exhibit multiple Gp60 alleles than humans

The stratified analysis revealed that cattle are substantially more likely than humans to harbour multiple Gp60 alleles (approximately 2.3-fold higher odds), which is likely to reflect a greater Gp60 subtype polyclonality rate. Inference is strengthened by the persistence of the host effect after adjustment for continent, sequencing depth, and Gp60 family in a multivariable model, indicating that the contrast is not readily explained by measured confounding. An association was observed between polyclonality and major-allele length, with longer alleles exhibiting increased odds of within-sample diversity. This effect was independent of host: neither a per-continent Mann–Whitney U test nor an OLS model adjusting for host, continent, and their interaction detected any meaningful difference in major-allele length between humans and cattle (Europe: $p=0.60$, North America: $p=0.95$, OLS $p=0.48$, adjusted $R^2=-0.001$). This length effect is biologically plausible, as longer STRs are more prone to slipped-strand mispairing during replication. However, because the generation of PCR slippage artefacts share a similar mechanistic origin, distinguishing biological from technical sources of variation in longer alleles is non-trivial. Accordingly, the observed association should be interpreted with caution, and future studies leveraging high-fidelity amplification or long-read sequencing may help disentangle these effects.

Because estimates of multiplicity at STR loci are sensitive to artefactual alleles, a conservative, pre-specified filtering scheme was adopted to suppress PCR stutter. An *L*1 near 0.15 is widely used in forensic STR work [28, 29], although lower values are sometimes chosen [27, 30]. Importantly, those thresholds were developed for fragment sizing rather than NGS allele frequencies, so they should be treated as heuristic. To assess sensitivity, we performed an exploratory sweep over allele-count and *L*1 values. The cattle-human contrast remained directionally stable across a neighbourhood around the pre-specified settings, whereas very lenient filters attenuated the signal in a manner consistent with rising stutter noise rather than a biological reversal. We therefore base formal conclusions on the pre-specified threshold set and view the sweep as a robustness check rather than a multiple-comparison exercise.

The elevated polyclonality observed in cattle, combined with the association between major allele length and polyclonality, suggests two non-exclusive mechanisms underlying within-host diversity at the Gp60 locus:

1. **In-host diversification** via slipped-strand mispairing, which generates novel alleles during replication. This mechanism is supported by the observed positive association between major-allele length and polyclonality, consistent with the known propensity of longer STR tracts to undergo slippage.
2. **Superinfection** from multiple sources, resulting in the co-occurrence of genetically distinct Gp60 alleles within the same host. This is supported by the markedly higher polyclonality rates observed in cattle, which are likely driven by intense transmission pressure in agricultural settings, high environmental oocyst burdens, and repeated exposure via shared water or fomites.

Together, these findings point to both de novo generation of allelic variants and repeated exogenous introduction as contributors to Gp60 polyclonality. From a public-health perspective, frequent mixed infections in livestock may increase the risk of zoonotic spillover and facilitate genetic exchange between divergent lineages, underscoring the importance of targeted surveillance at the animal–human interface.

Gp60 subtyping interrogates a single hypervariable locus, and consequently mixed infections detected here may under-represent genome-wide diversity. Longitudinal sampling and whole-genome assays will be important to confirm the host contrast, quantify within-host dynamics, and determine whether polyclonality in cattle reflects repeated exposure, prolonged carriage, or enhanced within-host diversification.

### Allelic associations, host plasticity, and geographic structure

Analysis of allele co-occurrence among polyclonal samples, conditioned on continent of origin, revealed both positive and negative associations (Fig. 6). Several positively associated pairs differed by a single repeat unit, consistent with recent divergence via replication slippage or co-transmission under high-exposure conditions. In contrast, multiple allele pairs exhibited statistically significant mutual exclusivity, including cross-family exclusions (e.g., A5G3-IIc with A15G1R1-IIa). These were detected within continents, suggesting that geographic segregation alone cannot explain the pattern. The most prevalent allele, A15G2R1-IIa, showed widespread exclusion of other alleles, likely reflecting a combination of high baseline frequency and genuine competitive incompatibility. These patterns could arise from within-host competition (e.g., differential fitness, immune evasion, or niche occupancy) or from biased introduction histories; distinguishing these mechanisms will require replication at additional loci, whole-genome metrics, and functional follow-up.

While artefactual contributions from PCR stutter cannot be fully excluded, filtering parameters and robustness checks across a broad threshold sweep support the qualitative validity of these findings. Nonetheless, because PCR stutter and slipped-strand mispairing can produce indistinguishable one-repeat variants at the Gp60 locus, complete removal of stutter-derived artefacts cannot be guaranteed. Consequently, caution is warranted when interpreting associations involving near-neighbour repeat variants due to the possibility of residual stutter artefacts.

Subtype distribution patterns were broadly consistent with established host associations and geographic trends. A15G2R1-IIa was the most frequently detected allele across the dataset, predominating in human and small ruminant samples, and aligning with its previously reported global ubiquity and non-host specificity [31–33]. In contrast, A19G1-IId dominated cattle samples from Asia, although this signal likely reflects sampling bias, as all Asian sequences originated from Chinese cattle. North American and European samples exhibited considerable overlap in subtype composition but also included region-specific alleles such as A15G2R2-IIa in North America and A17G1R1-IIa in Europe. A subset of alleles traditionally regarded as host-restricted displayed unexpected distributions. Notably, A19G2R1-IIa, previously described as cattle-associated [34], was recovered from 12 human samples in this dataset. These findings indicate that certain Gp60 subtypes may exhibit broader host plasticity than previously recognised, and underscore the value of continued genomic surveillance to track potential shifts in host adaptation.

### MOI may act as a ‘genetic crucible’ for diversification

Across continents, A15G2R1-IIa was widespread in humans and small ruminants, whereas A19G1-IId dominated cattle samples taken in China. The detection of IIa/IIc co-infections in North American humans and IId co-existing with a *C. tyzzeri* IXa-like allele in a mouse highlights the potential for subtypes, subspecies, and even entirely different species with different host affinities to undergo recombination through sexual reproduction. We hypothesise that such mixed infections, a necessary prerequisite for recombination, may act as a *genetic crucible*, facilitating diversification through within-host recombination. Such recombinant populations of *Cryptosporidium spp.* are likely to present substantial challenges in the control of this parasite worldwide. These results warrant an investigation into the association between MOI and recombination on larger scale, multi-continent datasets, and using multiple markers or genomics to robustly test this hypothesis.

### Technical limitations of BlooMine

The primary limitation of BlooMine is its reliance on the complete query (target sequence + flanking regions) being captured by enough reads to carry out MOI analysis. Larger query regions will require larger read sizes which are not produced by illumina sequencing platforms. However, the advent and employment of long-read technologies will facilitate the analysis of longer and more complex repeat rich regions, with which polyclonality may be characterised. Future work on BlooMine will support the analysis of formats commonly utilised by these long-read sequencing platforms.

## Conclusion

Polyclonality in *Cryptosporidium parvum* shapes transmission, host adaptation, and diversification. Continent-stratified analyses indicate cattle have higher odds of multi-subtype Gp60 infections, consistent with the hypothesis that intensive agriculture elevates transmission and, in turn, polyclonality. Patterns of allele co-occurrence may suggest rapid STR-mediated microevolution, while mutual exclusivity between certain alleles points to possible competitive exclusion. Some subtypes exhibited unexpected host distributions, challenging assumptions about host restriction. These findings raise the hypothesis that elevated polyclonality may act as a genetic crucible, facilitating within-host recombination and subtype diversification. This hypothesis is consistent with the observed allele diversity and co-occurrence patterns we report here, and should be rigorously explored in future multi-locus or whole-genome studies. By enabling high-resolution, alignment-free detection of STR variation from NGS data, BlooMine provides a scalable tool for investigating within-host diversity and provides a framework for future MOI surveillance and evolutionary studies.

## Supplementary Information

Supplementary Material 1

## Data Availability

The source code for BlooMine is available open source at https://github.com/ArthurVM/BlooMine. Supplementary data, metadata for samples included in this analysis, BlooMine reports, filtering scripts, and python notebooks containing code used in analysis can be found at https://github.com/ArthurVM/BlooMine_paper_supplementary.

## Abbreviations

AF: Allele frequency
BD: Breslow–Day test for homogeneity of stratum-specific odds ratios
CI: Confidence interval
CMH: Cochran–Mantel–Haenszel test / pooled analysis across strata
DNA: Deoxyribonucleic acid
FASTQ: Text-based format for sequencing reads (per-base calls and quality)
INDEL: Insertion/deletion variant
IQR: Interquartile range
k-mer: Substring of length *k* used in sequence analysis
MH OR (a.k.a. OR_MH, ORMH): Mantel–Haenszel pooled odds ratio across strata
MOI: Multiplicity of infection
NGS: Next-generation sequencing
OLS: Ordinary least squares (regression)
OR: Odds ratio
PCR: Polymerase chain reaction
SNP: Single nucleotide polymorphism
SPaln: Second-pass pseudo-alignment step in BlooMine
STR: Short tandem repeat (microsatellite)
VNTR: Variable-number tandem repeat
WGS: Whole-genome sequencing

## Gene/locus shorthand

Gp60: 60 kDa surface glycoprotein gene used for Cryptosporidium subtyping; alleles denoted as patterns such as A15G2R1 (counts of TCA, TCG, and terminal ACATCA motifs in *C. parvum*), with family label (e.g., A15G2R1-IIa in this paper; IIaA15G2R1 conventionally when molecular typing is carried out)
